# Betaine, a beneficial mimetic for long-term exercise

**DOI:** 10.1093/procel/pwaf057

**Published:** 2025-07-08

**Authors:** Xianhong Ji, Zi-Yu Wei, Pan Yang, Xiaoqiang Tang, Hou-Zao Chen

**Affiliations:** Department of Biochemistry and Molecular Biology, State Key Laboratory of Common Mechanism Research for Major Diseases, Institute of Basic Medical Sciences, Chinese Academy of Medical Sciences and Peking Union Medical College, Beijing 100005, China; Department of Biochemistry and Molecular Biology, State Key Laboratory of Common Mechanism Research for Major Diseases, Institute of Basic Medical Sciences, Chinese Academy of Medical Sciences and Peking Union Medical College, Beijing 100005, China; Department of Biochemistry and Molecular Biology, State Key Laboratory of Common Mechanism Research for Major Diseases, Institute of Basic Medical Sciences, Chinese Academy of Medical Sciences and Peking Union Medical College, Beijing 100005, China; Key Laboratory of Birth Defects and Related Diseases of Women and Children of MOE, State Key Laboratory of Biotherapy, West China Second University Hospital, Sichuan University, Chengdu 610041, China; National Health Commission Key Laboratory of Chronobiology, Sichuan University, Chengdu 610041, China; Development and Related Diseases of Women and Children, Key Laboratory of Sichuan Province, West China Second University Hospital, Sichuan University, Chengdu 610041, China; Department of Biochemistry and Molecular Biology, State Key Laboratory of Common Mechanism Research for Major Diseases, Institute of Basic Medical Sciences, Chinese Academy of Medical Sciences and Peking Union Medical College, Beijing 100005, China; Medical Epigenetics Research Center, Chinese Academy of Medical Sciences, Beijing 100005, China

**Figure 1.**
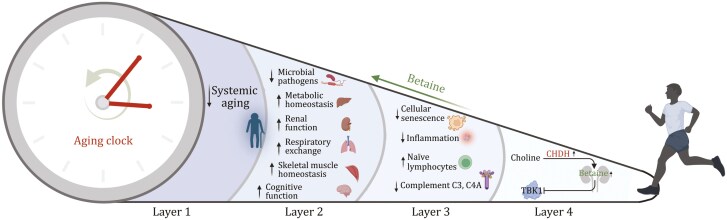
Long-term exercise (LE) reverses the aging clock by stimulating the release of betaine from the kidneys. LE provokes betaine synthesis in kidney by increasing choline dehydrogenase (CHDH) expression, then betaine inhibits inflammation by binding to TBK1 (Layer 4). These biomolecular events reduce cellular senescence, inflammation level, and Complement C3 and C4A, but also increase Naïve lymphocytes at the cellular level (Layer 3). With improved cellular function, LE ameliorates metabolic stress and cognitive impairment and, at the same time, improves renal, respiratory, and muscular functions (Layer 2). Overall, it slows down systemic aging and reverses the aging clock (Layer 1). The figure was created using BioRender.com.

What happens to your body when you are exercising? This fundamental question has fascinated scientists for a long time. It is common sense that habitual physical activity promotes good health, maximizes lifespan, and improves wellness (Sawalla Guseh et al., 2022; Sun et al., 2023b). There is some pioneering work suggesting that physical activity can alter the metabolite signature (Li et al., 2022) and increase cardio-pulmonary fitness (Nayor et al., 2022). However, the molecules that transduce exercise-mediated benefits remain a missing piece of the puzzle of sports medicine, which is crucial for developing anti-aging strategies. Recently, Geng and colleagues presented an exciting study demonstrating that regular exercise induces betaine to rejuvenate systemic aging in mammals (Fig. 1) (Geng et al., 2025).

To our knowledge, this is the first research to probe exercise mimetic systematically. First, 13 healthy males were enrolled based on the inclusion–exclusion criteria. They were required to undergo a 45-day rest period, after which they followed a 25-day running program with a stringent diet. Second, their blood and fecal samples were collected before and after exercise. To distinguish adaptations to Long-term Exercise (LE) from the immediate response of Acute Exercise (AE), phlebotomy from LE was postponed to 24 hours compared with instant sampling in AE. Then, specimens were subjected to multi-omics approaches, including single-cell transcriptional profiling of peripheral blood cells, plasma proteome, untargeted metabolomics from feces and plasma, microbiome, and Olink inflammation panel. After data integration and enrichment analysis, they discovered a low intra-category association among datasets generated from the AE group, with little mechanistic insight. In contrast, a strong association implicated in the LE group led to an explicable hypothesis, where betaine metabolism has emerged as the top-most adaptation both in organ metabolomes and transcriptomes, indicating betaine a vital molecule maneuvering a provoked coordination across 4 layers of aging. Betaine binds to and inhibits TANK binding kinase 1 (TBK1) that exacerbates inflammation during aging. Finally, they found that betaine administration protects against aging across various organs by reducing senescence-related markers and inflammatory factors.

The highlight of this study is the application of multi-omics. Unlike Qu and colleagues, who performed exhaustive search to find molecular mimetics (Qu et al., 2025), the integration of omics yields robust results. Integration in acute exercise do not contain hubs connecting different layers of mechanisms. While “more proportions of metabolites and plasma proteins” emerged as central factors across multiple layers from the peripheral blood cell transcriptome to the plasma metabolome, this indicates systemic remodeling and cross-layer coordination after long-term exercise. This study opens the door to a new paradigm of mimetic discovery, in the case of exercise mimetic, namely betaine.

Well, what is betaine biochemically? It is also called trimethylglycine, which is obligatory in diet due to insufficient synthesis from free choline by choline dehydrogenase (Dobrijević et al., 2023), and its active engagement in the methionine cycle, primarily serving as a methyl donor and an osmolyte (Zawieja and Chmurzynska, 2025). Luckily, it is abundant in dietary supplements, such as beets, wheat bran, and spinach, which can be easily absorbed by our intestines through various transporters.

This molecule has well-recognized benefits, including anti-inflammatory effects, cardiovascular protection, and the prevention of senescence. Betaine supplements break down homocysteine, which ignites pyroptosis in macrophages and causes many other age-related health problems, for example, atherosclerosis, hypertension, and cognitive impairment such as dementia (Zawieja and Chmurzynska, 2025). Besides, it functions as an osmolyte to retain water and reduce hyperosmotic stress, which enhance protein thermostability (Qu et al., 2025), and ensure a wide range of physiological process involving macromolecular machines (Akabayov et al., 2013; Ling et al., 2023). It could also accommodate the microbial community Geng et al., (2025) , probably for symbiosis. Additionally, this study reveals that betaine can bind to TBK1 to manifest anti-aging effects. In all, betaine is represented as a pivotal molecule that safeguards against aging across four scales Zhang et al., (2015), which inherently connects malfunctioning cell components, tissue deterioration, systemic dysfunction and organismal aging.

Then, where does betaine come from? This is an intriguing question that worth a second look. Neither betaine nor its precursor, choline, increases in human fecal metabolites, therefore, it is reasonable to suggest an endogeneous source since all subjects stick to a tailored diet, and no microbial enzymes responsible for its production. But from which organ? By profiling different organs using single-cell transcriptomics, the authors have identified a remarkable adaptation in the rodent kidney, followed by the muscles, the aorta, and the liver. While analyzing metabolites, they found striking conservation in the kidney, followed by the lungs, the adrenal gland, and skeletal muscles, all of which can exchange betaine through circulation. Specifically, betaine accumulation is crucial for kidney osmoprotection, which highlights the pivotal role of renal system in long-term benefits of exercise.

Overall, this study inspired us with betaine’s therapeutic values. For researchers interested in different regimens, a pipeline has established. By including more features, such as gender and adiposity,  they would be expected to find more valuable metabolites that conscripted into different workout routines. For drug innovators, who used to hinge on incretin mimetics, like Semaglutide, Tirzepatide and recently Mazdutide, which have exhibited cardiovascular benefits while losing weight, betaine was found completely outside the box. Similar to their previous study (Sun et al., 2023a), betaine, alongside ascorbic acid, improves physical performance and cognitive functions in rodents (Geng et al., 2025). So, can betaine be a pill to win out in the next round by offering extensive benefits such as optimizing energy partitioning, building up muscles, and improving overall well-being? Evidence is needed to back up. For the elderly who are disabled and physically limited, exercise or not is a Hobson’s choice, but now betaine might come to help.
